# Comparative Studies on the Induction of *Trichoderma harzianum* Mutanase by α-(1→3)-Glucan-Rich Fruiting Bodies and Mycelia of *Laetiporus sulphureus*

**DOI:** 10.3390/ijms13089584

**Published:** 2012-07-31

**Authors:** Adrian Wiater, Małgorzata Pleszczyńska, Janusz Szczodrak, Grzegorz Janusz

**Affiliations:** 1 Department of Industrial Microbiology, Maria Curie-Skłodowska University, Akademicka 19, 20-033 Lublin, Poland; E-Mails: mplesz@poczta.onet.pl (M.P.); szczo@poczta.umcs.lublin.pl (J.S.); 2 Department of Biochemistry, Maria Curie-Skłodowska University, Akademicka 19, 20-033 Lublin, Poland; E-Mail: g.gjanusz@gmail.com

**Keywords:** *Laetiporus sulphureus*, mutanase, *Trichoderma harzianum*, α-(1→3)-glucan, fruiting body, polypore fungus

## Abstract

Mutanase (α-(1→3)-glucanase) is a little-known inductive enzyme that is potentially useful in dentistry. Here, it was shown that the cell wall preparation (CWP) obtained from the fruiting body or vegetative mycelium of polypore fungus *Laetiporus sulphureus* is rich in α-(1→3)-glucan and can be successfully used for mutanase induction in *Trichoderma harzianum*. The content of this biopolymer in the CWP depended on the age of fruiting bodies and increased along with their maturation. In the case of CWP prepared from vegetative mycelia, the amount of α-(1→3)-glucan depended on the mycelium age and also on the kind of medium used for its cultivation. All CWPs prepared from the individually harvested fruiting body specimens induced high mutanase activity (0.53–0.82 U/mL) in *T. harzianum* after 3 days of cultivation. As for the CWPs obtained from the hyphal mycelia of *L. sulpureus*, the maximal enzyme productivity (0.34 U/mL after 3 days of incubation) was recorded for CWP prepared from the 3 week-old mycelium cultivated in Sabouraud medium. Statistically, a high positive correlation was found between the total percentage content of α-(1→3)-glucan in the CWP and the mutanase activity.

## 1. Introduction

Mutanase, α-(1→3)-glucan 3-glucanohydrolase, catalyzes hydrolysis of α-(1→3)-glucoside linkages in streptococcal mutans (water-insoluble and highly branched glucans present in oral biofilms), and α-glucans building the structure of the fungal cell wall. The mutan-degrading enzyme has a high potential as a caries preventive agent, due to its ability to solubilize mutans present in dental plaque as well as denture plaque [1–3]. As an active ingredient in dentifrice preparations and chewing gum, mutanase could become a useful supplement to mechanical cleaning of teeth and dentures with a toothbrush, dental sticks, and dental floss. In addition to their potential usefulness in dentistry as an oral therapeutic agent, α-(1→3)-glucanase might be applicable to investigations of α-(1→3)-glucosidic linkages occurring in fungal cell-wall structures and glucans of certain higher plants. Mutanase obtained in a pure form is a unique tool for studying the chemical structures of these carbohydrates. In addition, the isolation of mutanase gene from the biocontrol agent and their further transfer to a plant genome can result in significant improvement in plant defence against fungal pathogens. For example, *Arabidopsis thaliana* containing the α-(1→3)-glucanase gene cloned from *T. harzianum* shows significant resistance to infection by the foliar pathogen *Botrytis cinerea* [4]. Moreover, various preparations of mutanase have also been successfully used for obtaining fungal protoplasts, improvement of filterability of wine obtained from a grapes infected with the mould *B. cinerea*, and improvement of filterability and injectability during the secondary and tertiary petroleum recovery by waterflooding [5–7].

The mutanase reported until recently has been inducible in media containing streptococcal mutan. Unfortunately, this biopolymer is not available in bulk quantities due to the pathogenicity of its producers (cariogenic streptococci), the necessity to use complex and expensive media (e.g., beef brain-heart infusion), the multistage production process, low product yields (no more than 2 g/L), and high structural heterogeneity. Substitution of mutan by more available inducers could facilitate commercial-scale mutanase production. An alternative source of α-(1→3)-glucans is the fungal cell wall. We have shown that the cell wall preparation from *Laetiporus sulphureus* effectively induced mutanase in *T. harzianum* [8] and, to the best of our knowledge, this was the first occasion when this material had been used for mutanase production.

*L. sulphureus* is a polypore fungus belonging to a specific group of wood-decomposing *Basidiomycetes* growing on deciduous trees. This species is widely distributed in Europe, Asia, and North America [9]. It produces large and strong fruiting bodies which are edible when young, and whose wet biomass sometimes reaches a few kilograms. Moreover, this fungus has long been used in Asian herbal medicine [10] and is also known as a source of antioxidant, antimicrobial, cytostatic and immunostimulative agents and a producer of HIV-1 reverse transcriptase inhibitors [11–13].

*L. sulphureus* can also be cultivated on a larger scale in a laboratory both as fruiting bodies in a solid-state surface culture and as a hyphal mycelium in a fermenter submerged culture [14–15]. Moreover, the cell wall of this fungus is the richest source of α-(1→3)-glucans (up to 78% dry matter) [16]. Therefore, a mycelial material of *L. sulphureus* could represent an inexpensive, easily available, and a human-safe source of an inducer for mutanase production.

The aim of this study was estimation of the α-(1→3)-glucan content in the cell wall and possibilities for application of the harvested fruiting bodies and cultured mycelium of the polypore fungus *L. sulphureus* as *T. harzianum* mutanase inducers.

## 2. Results and Discussion

It is well known that the best mutanase inducer is α-(1→3)-glucan synthesized by cariogenic streptococci or isolated from fungal cell wall [17]. In our previous study, we demonstrated that *L. sulphureus* fruiting bodies were an excellent source of these inducers [8,18].

Among the three tested preparations, *i.e.*, lyophilized and milled preparation of fresh fruiting bodies of *L. sulphureus*, cell wall preparation (CWP), and purified α-(1→3)-glucan preparation, the highest mutanase productivity (0.71 U/mL) was obtained on CWP, which was also the best reported in the literature [8]. The use of this inducer is further justified from the economic point of view, since its preparation requires a simple and inexpensive procedure.

However, the CWP from *L. sulphureus* used for mutanase induction in our previous studies was obtained from a mixture, representing a combination of young and aged basidiocarps. The composition of the preparation was not homogenous, as it contained α-(1→3)-glucan fractions originating from many specimens; hence, the induced mutanase activity resulted from the action of various α-(1→3)-glucans. Therefore, in the present work, individual fruiting bodies were analysed for the amount of α-(1→3)-glucan and its effect on the synthesis of *T. harzianum* CCM F-340 mutanase. Fruiting bodies of *L. sulphureus* (16 specimens) harvested from various hosts at different times and in many locations were described and divided into three groups distinguished by their maturity, *i.e.*, an immature fruiting body without the hymenophore (A_1_–A_5_), a mature fruiting body with immature spores (B_1_–B_3_) and a mature fruiting body with mature spores (C_1_–C_8_) (Table 1). This classification was consistent with the amount of α-(1→3)-glucan in the *L. sulphureus* cell wall, which, as suggested by Jelsma and Kreger [16], increases along with fruiting body maturation. Our results are in good agreement with these authors. It was found that the amount of α-(1→3)-glucan depended, to a certain extent, on the age of the fruiting bodies. In young specimens (group A), their amount oscillated between 17.3% and 37.6% of dry weight; in group B, these polysaccharides were present at a concentration of 36.5–41%, whereas in the oldest ones (group C), they ranged from 42.8 to 47.8% of dry weight.

The CWPs from the harvested fruiting body specimens were used as inducers of *T. harzianum* mutanase production (Table 2). All the samples described induced high mutanase activity (at a level of 0.53 to 0.82 U/mL) after 3 days of submerged cultivation in shaken flask cultures. The highest enzyme productivity was obtained on the CWP from specimen C_3_, and it was by 15% higher than that (0.71 U/mL) obtained on the mixture of various fruiting bodies of *L. sulphureus* used in the previous studies [8]. Moreover, currently this is the best-reported productivity in the literature. For example, while testing enzyme production by *T. harzianum* OMZ 779 in fermenter runs, Guggenheim and Haller [19] obtained an activity of 0.08 U/mL after 155 to 165 h. In shaken flask cultures supplemented with 1% mutan, mutanase activity reached its maximum yield of 0.16 U/mL after 120 h of incubation. Meanwhile, using the same fungal strain, Quivey and Kriger [20], reached the specific mutanase activity of 0.37 U/mg protein after 4 days in shaken flask cultures. Also, based on *Streptomyces chartreusis*, Inoue *et al.* [21] obtained the maximum mutanase activity of 0.005 U/mL after 3 days in shaken flask cultures. In the case of bacterial mutanases, Pleszczyńska *et al.* [22] achieved enzyme activities of 0.35 U/mL and 1.17 U/mg protein in a flask culture of *Paenibacillus* sp. MP-1 grown for 48 h on CWP from fruiting bodies of *L. sulphureus*. Meyer and Phaff [23] obtained up to 0.31 U/mL from *Bacillus circulans* WL-12 by supplementing the media with whole cells of *Schizosaccharomyces pombe* or purified α-(1→3)-glucan from *Aspergillus niger. Streptomyces* KI-8 produced mutanase (0.16 U/mL) when it was cultured on α-(1→3)-glucan isolated from dried fruiting bodies of *Lentinus edodes* [24].

**Figure 1:**
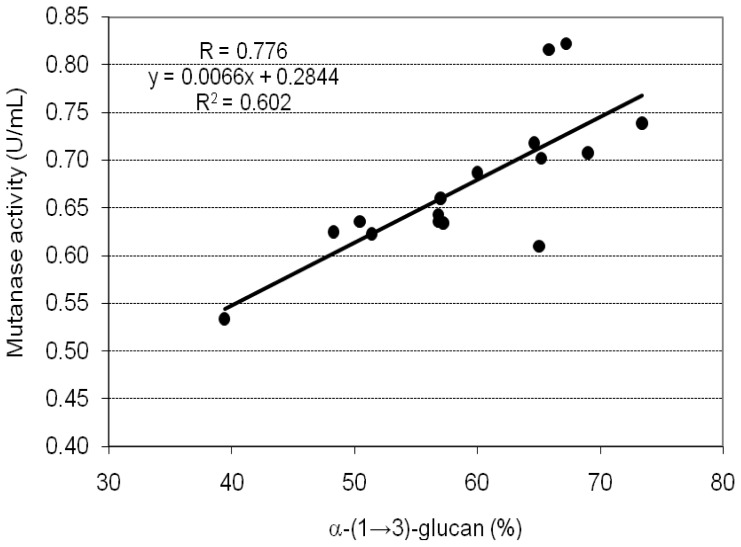
Relationship between the mutanase activity obtained on particular CWP from fruiting bodies of *L. sulphureus* used as enzyme inducers and the content of α-(1→3)-glucan in each of these preparations. Data obtained from statistical analysis: Pearson correlation (*R*), determination (*R^2^*) and linear regression (*y*), *p < 0.05*.

As can be seen from the data summarized in Table 2, mutanolytic activity higher than 0.71 U/mL was also obtained on the CWPs from specimens C_1_, C_2,_ and C_5_. Moreover, the level of enzyme activity obtained on CWP from the individual specimens was closely related to the amount of α-(1→3)-glucan in the inducer. The correlation was confirmed by statistical analyses (Figure 1). The data obtained from those analyses (Pearson’s correlation coefficient *R* = 0.776, the coefficient of determination *R^2^* = 0.602, linear regression *y* = 0.0066*x* + 0.2844, *p* < 0.05) indicate a high positive correlation between the content of α-(1→3)-glucan in the CWP and the induced mutanase activity. However, no such correlation was observed after assessment of the enzyme activity per gram of α-(1→3)-glucan used for induction (Table 2). For example, CWP with the lowest content of α-(1→3)-glucan (A_1_) induced the highest enzymatic activity (3.38 U/g of α-(1→3)-glucan), whereas CWP with the highest content of α-(1→3)-glucan (C_5_) induced one of the lowest activities (2.51 U/g of α-(1→3)-glucan). Based on these results, it can be supposed that the level of mutanase production depends not only on the amounts of α-(1→3)-glucan, but also on other factors such as the spatial arrangement of linkages in the inducer molecule and the accessibility of enzyme to α-(1→3) sequences.

**Figure 2:**
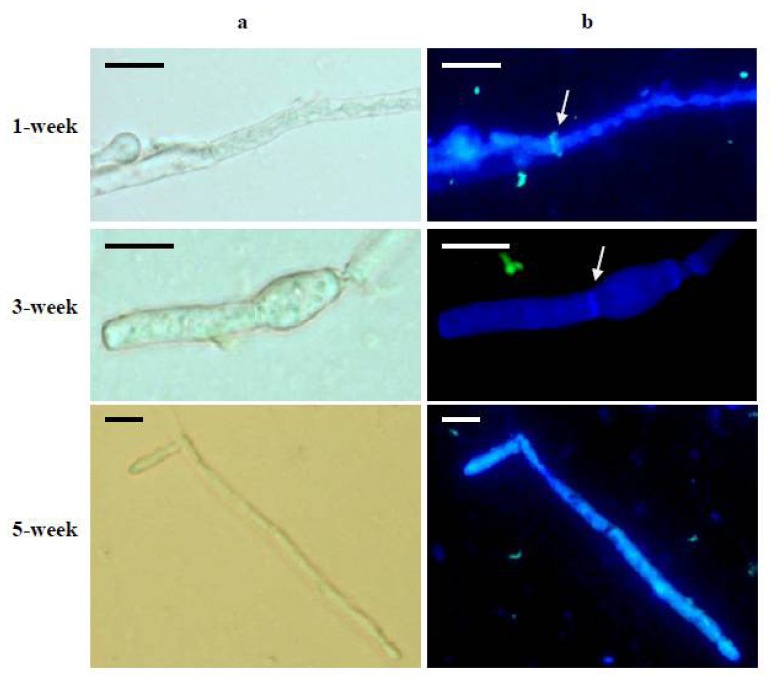
Localization of α-(1→3)-glucan in hyphae of *L. sulphureus* CBS 388.61 cultured on medium IV by means of fluorophore-labeled antibodies. (**a**) hyphae in the light microscopy; (**b**) fluorescent image of the same hyphae. Arrows indicate the accumulation of glucan in the hyphal septum. Twenty samples were observed and typical images are presented. Scale bar = 20 μm.

An attempt was also made to select a culture medium for the production of *L. sulphureus* mycelium and to investigate the ability of mycelial biomass to induce mutanase. Hyphal mycelium of *L. sulphureus* can be readily obtained on a large scale. Consequently, mutanase production could become independent of the fruiting bodies, which can be harvested only over limited periods (late spring and early summer). In the experiments described, the strain *L. sulphureus* CBS 388.61 was cultivated in nine media for up to 5 weeks. The cultures were withdrawn at appropriate intervals (1, 3, and 5 weeks) and the mycelium biomass was measured. We used specific fluorophore-labeled antibodies in order to detect α-(1→3)-glucan in the cell wall of particular mycelium samples. Figure 2 shows a typical image of this staining (*L. sulphureus* mycelium cultured on medium IV), where α-(1→3)-glucan is clearly labeled in the hyphae. Moreover, a significant accumulation of glucan was observed in hyphal septa.

As shown in Table 3, five culture media yielded over 1 g/L of dry weight of mycelium after 5-week incubation. The maximum biomass production (5.53 g/L) was achieved in Sabouraud medium (IV) after 5-week cultivation. The mycelium production yields in media V, VI, VII, and IX were relatively low; therefore, they were excluded from further study.

The content of α-(1→3)-glucan in water-extracted mycelium (CWP) and non-treated mycelium obtained after 1- to 5-week incubation in 5 selected media was also analyzed (Tables 3, 4). It was found that a 3-week mycelium obtained in Sabouraud medium (IV) contained the highest amount of α-(1→3)-glucan, *i.e.*, 19.7% and 27.8% in non-treated mycelium and CWP, respectively. Apart from one case (specimen A_1_), the amount of α-(1→3)-glucan in mycelia of *L. sulphureus* was significantly lower (by ca. 7.2–28.1% and 11.2–45.6% for non-treated mycelium and CWP, respectively) than that isolated from a majority of the fruiting bodies. In general, the content of α-(1→3)-glucan in the mycelia obtained in the nine various media increased and exhibited the highest value after 5-week incubation (Table 3). However, in the case of media IV and VIII, the amount of this biopolymer decreased compared to the 3-week mycelium, which may be explained by cell wall autolysis that starts after exhaustion of external carbon sources and reserve substrates. During this process, lytic enzymes (among others α-(1→3)-glucanase) with activities hydrolyzing the polymers contained in the fungal cell wall are released [25,26]. The fluctuations of mycelium dry weight were also reported by Jaroszuk-Œciseł *et al.* [27] for *Fusarium culmorum* cultivated in a glucose medium. After 42-day incubation, the percentage decrease in the dry weight of mycelium in relation to its maximum dry weight amounted to *ca.* 20%.

**Figure 3:**
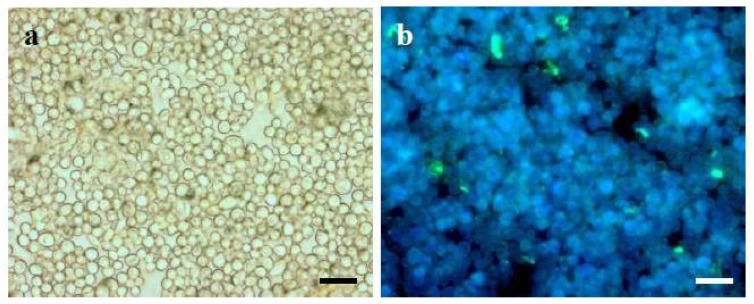
Visualization of spores and spore wall α-(1→3)-glucan in 5-week biomass of *L. sulphureus* CBS 388.61 cultured on medium IV. (**a**) view in light microscopy; (**b**) fluorescent image of the same view; α-(1→3)-glucan detected by means of fluorophore-labeled antibodies. Twenty samples were observed and typical images are presented. Scale bar = 20 μm.

On the other hand, the cause of the reduction in the amount of α-(1→3)-glucan in the mycelium of *L. sulphureus* may have been the production of spores, whose considerable amounts were detected in the 5-week biomass obtained in medium IV (Figure 3). A study by Grün *et al.* [28] indicated that the synthase for vegetative cell wall α-(1→3)-glucan (Ags1p) is downregulated during sporulation, whereas Ags1p homologs for spore wall α-(1→3)-glucan are upregulated. It should be emphasized here that during preparation of CWP from the 5-week *L. sulphureus* mycelium, the spores were washed away as a result of biomass pre-rinsing and filtering. This resulted in a lower content of α-(1→3)-glucan in the CWP.

**Figure 4:**
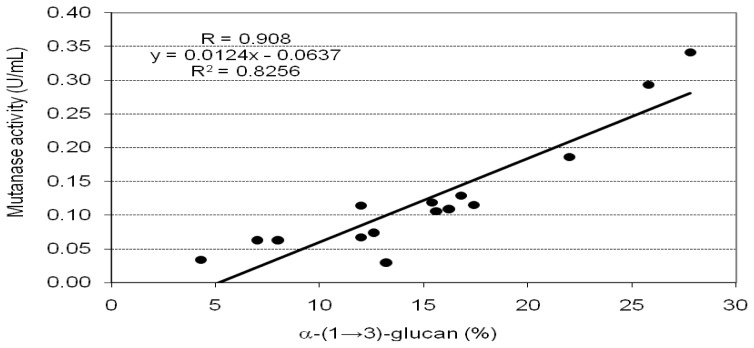
Relationship between the mutanase activity obtained on particular CWP from mycelia of *L. sulphureus* CBS 388.61 used as enzyme inducers and the content of α-(1→3)-glucan in each of these preparations. Data obtained from statistical analysis: Pearson correlation (*R*), determination (*R^2^*) and linear regression (*y*), *p < 0.05*.

The effect of CWPs from mycelia on mutanase production by *T. harzianum* CCM F-340 is shown in Table 4. Among the five media used in this stage of the research, the CWPs from the mycelium obtained on Sabouraud medium (IV) were the most effective with respect to mutanase productivity. The maximum mutanase yield (0.34 U/mL) was monitored on the CWP from the three-week mycelium. However, it was still about 2.5 times lower than the best activity induced by the CWP derived from fruiting body C_3_. Nevertheless, it is noteworthy that CWPs derived from both, fruiting bodies and mycelia are in many cases equally efficient as *Trichoderma* mutanase inducers, especially if we relate the level of enzyme activity to gram of α-(1→3)-glucan content in fungal material (Tables 2, 4). As in the case of induction using the CWP from fruiting bodies, in the case of the CWP from mycelia, we observed a high positive value of the Pearson correlation coefficient (*R* = 0.908, *p* < 0.05) and the linear correlation (*y* = 0.0124*x* − 0.0637, *R^2^* = 0.8256, *p* < 0.05) indicated close dependence between the content of α-(1→3)-glucan in CWP and the induced mutanase activity (Figure 4).

The results from the present study have indicated that both the fruiting bodies and mycelium of *L. sulphureus* may be a valuable source of mutanase inducers. Furthermore, in terms of industrial production, the use of mycelia for efficient mutanase synthesis renders continuous production of the enzyme possible with no need to use seasonally growing fruiting bodies.

## 3. Experimental Section

### 3.1. Microorganisms

The fruiting bodies of *Laetiporus sulphureus* (Bull.: Fr.) Murrill were harvested from various host trees at different times and in many locations in Poland (Table 1). The specimens were identified by molecular biological analysis of the internal transcribed region (ITS) of the 5.8S rDNA as described below. Voucher specimens are deposited in the Department of Industrial Microbiology, Maria Curie-Skłodowska University, Lublin, Poland. *L. sulphureus* CBS 388.61 (CBS, Utrecht, The Netherlands) was cultivated in nine liquid media for production of the mycelium. *Trichoderma harzianum* strain CCM F-340 (Czech Collection of Microorganisms, Brno, Czech Republic) was used as a starting culture for mutanase induction by cell wall material from *L. sulphureus*.

### 3.2. *Laetiporus sulphureus* Cultivation

The culture of *L. sulphureus* CBS 388.61 was maintained on malt extract agar (MEA) slants (2% malt extract, 1.5% agar), stored at 4 *°*C and subcultured every month. The seed cultures were grown in 500 mL flasks containing 100 mL of the incubation medium at 25 *°*C on a rotary shaker incubator at 200 rpm for 7 days. The culture incubation media (500 mL) in 1 L flasks were inoculated with 5% (v/v) of the adequate seed culture and then cultivated for 5 weeks at 25 *°*C on a rotary shaker incubator at 150 rpm. The composition of the nine different incubation media was described earlier, *i.e.*, medium I by Lamer-Zarawska *et al*. [29]; medium II by Davoli *et al.* [14]; medium III by Kartal *et al.* [30]; medium IV (Sabouraud Liquid Broth) by Baltimore Biological Laboratory, USA; media V-VIII by Jaouani *et al*. [31]; medium IX by Sani *et al.* [32]. Samples of biomass taken after 1, 3, and 5 weeks were centrifuged at 17001 × *g* for 30 min. After repeated washing with distilled water and drying by lyophilization, the mycelia were used for further analysis.

### 3.3. *Trichoderma harzianum* Cultivation

Stock cultures of *T. harzianum* CCM F-340 maintained at 4 °C on potato dextrose agar slants were used for inoculations. Liquid medium A (pH 5.3), as described by Mandels *et al.* [33], supported by 0.4% of CWP (cell wall preparation form *L. sulphureus*), 0.05% proteose peptone, and 0.1% Tween 80 was used for mutanase production. Shaken cultures were performed in 500 mL conical flasks containing 100 mL of sterile medium. The flasks were seeded with conidia to a final concentration of about 2 × 10^5^ conidia/mL and placed on an orbital rotary shaker at 300 rpm and 30 °C for 3 days.

### 3.4. Cell Wall and α-(1→3)-Glucan Preparation

The cell wall preparation (CWP) as well as the extraction and purification of α-(1→3)-glucan from fruiting bodies and mycelia of *L. sulphureus* were performed according to the procedure described by Wiater *et al*. [8]. The lyophilized fungal material was milled and the resulting powder was treated with water at 121 °C for 1.5 h (× 3). The wall material was removed by centrifugation (17001 × *g* for 30 min) and freeze-dried (Cell Wall Preparation, CWP). To isolate the alkali-soluble fraction, the CWP was suspended in 1 M NaOH under constant stirring. After overnight-incubation at room temperature, the supernatant was neutralized with 1 M HCl. The insoluble fraction was collected by centrifugation, washed with water (× 3), and lyophilized to give a white powder (purified α-(1→3)-glucan). The polysaccharides obtained were analyzed by FT-IR and ^1^H NMR to confirm α-(1→3)-glucan purity (data not shown).

### 3.5. Mutanase Assay

The standard mutanase assay mixture contained 0.5 mL of 0.2% (w/v) a dextranase-pretreated mutan (DTM) in 0.2 M sodium acetate buffer (pH 5.5) and 0.5 mL of the suitably diluted enzyme solution. After 1 h incubation at 45 °C, the reducing sugars released were quantified by the Somogyi-Nelson’ method [34–35]. One unit of mutanase activity (U) was defined as the amount of enzyme hydrolyzing mutan to yield reducing sugars equivalent to 1 μmol of glucose/min, and expressed as units per mL of culture (U/mL). 1 U corresponds to 16.67 nkat.

### 3.6. Preparation of Dextranase-Pretreated Mutan (DTM)

Dextranase-pretreated mutan (DTM) was prepared (50 U of dextranase/mg of native mutan, pH 6.0, 37 °C, 3 × 24 h) as a substrate for mutanase activity. Native mutan was synthesized from sucrose with the use of a mixture of crude glucosyltransferases of cariogenic *S. sobrinus*/*downei* CCUG 21020 (The Culture Collection, University of Göteborg, Sweden) as described previously [36]. Dextranase of *Penicillium* sp. with an enzyme activity of 12.9 U/mg preparation was purchased from Sigma-Aldrich (St. Louis, MO., USA). The linkage structure of the native and the dextranase-pretreated mutan determined by ^1^H NMR showed that they were mixed-linkage α-(1→3) and α-(1→6) biopolymers with a greater proportion of α-(1→3) to α-(1→6) linkages, namely, 59.1 and 40.9 mol% for native mutan and 79.8 and 20.2 mol% for DTM, respectively.

### 3.7. Genomic DNA Isolation, Amplification of ITS Sequences, and DNA Sequencing

The extraction procedure followed the methods of Borges *et al.* [37] with minor modifications. Small amounts of fungal mycelium were suspended in a lysis buffer (4 mM spermidine, 10 mM EDTA, 100 mM NaCl, 0.5% SDS, 10 mM β-mercaptoethanol, 40 mM Tris-HCl, pH 8.0) and incubated at 65 °C for 40 min. After incubation, the samples were sequentially extracted with phenol and chloroform, centrifuged for 20 min at 10,000 × *g*, precipitated with ice-cold ethanol, washed with 70% ethanol, dried and redissolved in TE buffer (1 mM Tris-HCl, 100 mM EDTA, pH 8.0). The purity and concentration of the DNA samples were evaluated using ND-1000 (Nanodrop, USA). Polymerase chain reaction amplifications (PCR) followed the protocol of White *et al.* [38] in a final volume of 50 μL. The primers ITS1, ITS2, ITS3, and ITS4 were used for PCR amplification and sequencing of the internal transcribed spacers from the ribosomal genes. The reactions were performed in a TPersonal thermocycler (Biometra, Germany). Amplified PCR products were quantified by gel electrophoresis on a 1% agarose gel stained with ethidium bromide and purified by microfiltration using a Clean-up kit (A&A Biotechnology, Poland). Sequencing was performed by fluorescent dye-terminator chemistry with the automated sequencer ABI 3730 (Applied Biosystems Inc., USA) following the manufacturer’s instructions.

### 3.8. Immunofluorescent Labeling of α-(1→3)-Glucan

Fluorescently labeled antibodies were used to localize of the α-(1→3)-glucan within the spores and cell wall of *L. sulphureus* [39]. Fresh biomass of *L. sulphureus* on Lab-Tek II Chamber slides (Nunc, Rochester, USA) was fixed with 3% (v/v) formaldehyde solution in distilled water at 65 °C for 30 min. The fixed fungal cells were washed three times in PBS buffer (137 mM NaCl, 2.7 mM KCl, 8.1 mM Na_2_HPO_4_, 1.5 mM KH_2_PO_4_, pH 7.4) before being infiltrated by 1% (v/v) Tween 20 in PBS buffer (PBS-T). To detect the presence of the α-(1→3)-glucan, 150 μL solution of mouse IgM MOPC-104E (0.1 mg/mL in PBS buffer) (Sigma, St Louis, MO, USA) as the primary antibody and 150 μL Alexa Fluor 488 goat anti-mouse IgM (μ-chain specific) (0.1 mg/mL in PBS buffer) (Sigma, St. Louis, MO, USA) as the secondary antibody were used. The samples were incubated with primary antibodies overnight at 4 °C in a wet chamber. Incubation with secondary antibodies was performed as follows: 2 h in dark, at 37 °C. Before observation by fluorescent microscope (Olympus BX 51), the antibody-labeled cells were rinsed three times with PBS buffer. The α-(1→3)-glucan was observed using excitation wavelength at 470/500 nm and emission at 525/550 nm.

### 3.9. Statistical Analysis

Statistical analysis of the data was performed on three replicates from each treatment. Standard deviations between the values obtained in each experiment were less than 5%. Standard deviations were determined using Microsoft^®^ Excel 2000 (Microsoft Corp., Redmond, Washington, USA). The Pearson correlation coefficient (R), the determination coefficient (R^2^), and the linear regression (y) were determined (using Microsoft^®^ Excel 2000) to show the direction and strength of the relationship between the amount of α-(1→3)-glucan in CWP and the mutanase activities in the culture fluid of *T. harzianum* after induction by a particular inductor. Other methodological details are included in the legends of the Tables and Figures.

## 4. Conclusions

In conclusion, the *L. sulphureus* is a common, safe, and easily identified polypore fungus. It can be cultivated (as fruiting bodies or mycelium) or harvested from infected trees. The results obtained suggest that the high content of α-(1→3)-glucan makes the fruiting bodies as well as mycelium of *L. sulphureus* economic and efficient inducers for large-scale production of microbial mutanases. Moreover, it was found that mycelium used as an inducer was equally effective, which facilitates continuous production of the enzyme without the necessity to use seasonally growing fruiting bodies.

## Figures and Tables

**Table 1:** Characteristics of harvested fruiting bodies of *L. sulphureus*.

Specimen	GenBankaccession NO.	Geographicorigin	Host tree	Fruiting body sizeª	Fruiting body maturity^b^	α-(1→3)-Glucan(%)^c^
A_1_	HM245764	Lublin(51°13′N, 22°33′E)	*Prunus cerasus*	+	+	17.3 ± 0.8
A_2_	HM245765	Piaseczno(52°03′N, 21°00′E)	*Salix alba*	+++	+	26.9 ± 1.3
A_3_	HM245763	Łomża(53°10′N, 22°03′E)	*S. alba*	++	+	28.0 ± 1.2
A_4_	HM245772	Warszawa(52°13′N, 21°00′E)	*Salix caprea*	+++	+	36.2 ± 0.9
A_5_	HM245770	Lublin(51°13′N, 22°33′E)	*S. alba*	+	+	37.6 ± 1.1
B_1_	HM245771	Węgorzewo(54°12′N, 21°44′E)	*Quercus robur*	++	++	36.5 ± 0.5
B_2_	HM245766	Lublin(51°13′N, 22°33′E)	*S. alba*	+	++	37.0 ± 1.4
B_3_	HM245768	Gęś(51°42′N, 23°00′E)	*Prunus avium*	+++	++	41.0 ± 1.5
C_1_	HM245761	Lublin(51°13′N, 22°33′E)	*Fraxinus excelsior*	++	+++	42.8 ± 1.3
C_2_	HM245762	Drobin(52°44′N, 19°59′E)	*Salix chrysocoma*	++	+++	44.7 ± 1.9
C_3_	HM245759	Lublin(51°13′N, 22°33′E)	*S. chrysocoma*	++	+++	46.4 ± 1.8
C_4_	HM245760	Frampol(50°40′N, 22°40′E)	*S. alba*	+++	+++	46.7 ± 2.1
C_5_	HM245758	Kurów(51°23′N, 22°11′E)	*S. caprea*	+++	+++	46.9 ± 0.8
C_6_	HM245774	Bytów(54°10′N, 17°29′E)	*Q. robur*	++	+++	46.9 ± 1.7
C_7_	HM245767	Zielona(50°59′N, 22°40′E)	*P. cerasus*	+	+++	47.0 ± 1.1
C_8_	HM245773	Legionowo(52°24′N, 20°55′E)	*Robinia pseudoacacia*	++	+++	47.8 ± 0.7

a Fruiting body size: (+) < 20 cm, (++) 20–35 cm, (+++) > 35 cm;

b Fruiting body maturity: (+) immature fruiting body (lack of hymenophore), (++) mature fruiting body with immature spores, (+++) mature fruiting body with mature spores;

c Amount of α-(1→3)-glucan in fruiting body dry mass. Results are shown as mean ± SD of three independent experiments.

**Table 2:** Effect of cell wall preparation (CWP) from the *L. sulphureus* fruiting bodies on mutanase production by *T. harzianum* in shaken flask cultures a.

CWP		Mutanase activity	
	
Specimen	α-(1→3)-Glucan (%)	U/mL	U/g α-(1→3) Glucan
A_1_	39.4 ± 0.8	0.534 ± 0.025	3.388 ± 0.112
A_2_	50.4 ± 1.9	0.636 ± 0.028	3.155 ± 0.098
A_3_	48.3 ± 1.3	0.625 ± 0.013	3.235 ± 0.052
A_4_	56.8 ± 2.1	0.643 ± 0.009	2.830 ± 0.121
A_5_	57.2 ± 1.6	0.634 ± 0.030	2.771 ± 0.046
B_1_	56.8 ± 1.9	0.636 ± 0.007	2.799 ± 0.077
B_2_	57.0 ± 1.7	0.660 ± 0.015	2.895 ± 0.133
B_3_	60.0 ± 1.1	0.687 ± 0.025	2.863 ± 0.135
C_1_	64.6 ± 2.2	0.718 ± 0.015	2.779 ± 0.127
C_2_	65.8 ± 1.5	0.816 ± 0.019	3.100 ± 0.037
C_3_	67.2 ± 1.7	0.822 ± 0.023	3.058 ± 0.121
C_4_	65.2 ± 1.2	0.702 ± 0.005	2.692 ± 0.099
C_5_	73.4 ± 2.2	0.739 ± 0.029	2.517 ± 0.078
C_6_	69.0 ± 1.7	0.708 ± 0.013	2.565 ± 0.067
C_7_	65.0 ± 1.3	0.610 ± 0.017	2.346 ± 0.111
C_8_	51.4 ± 0.9	0.623 ± 0.015	3.030 ± 0.135

a Submerged cultures were performed on modified Mandels’ A medium with 0.4% CWPs in 500 mL conical flasks, each containing 100 mL of the medium. Initial medium pH was 5.3. Results are shown as mean ± SD of three independent experiments.

**Table 3:** Comparison of kinetics of mycelium growth and production of cell-wall α-(1→3)-glucan during shaken flask cultures of *L. sulphureus* CBS 388.61 in different media.

	Mycelium age					
	
	1-Week-Old		3-Week-old		5-Week-old	
			
Medium	Biomassyield (g/L)	α-(1→3)-Glucan (%)^a^	Biomassyield (g/L)	α-(1→3)-Glucan (%)^a^	Biomassyield (g/L)	α-(1→3)-Glucan (%)^a^
I	1.26 ± 0.054	4.7 ± 0.17	2.82 ± 0.097	11.0 ± 0.36	3.40 ± 0.096	12.4 ± 0.19
II	0.08 ± 0.004	2.3 ± 0.05	0.58 ± 0.020	5.8 ± 0.16	1.01 ± 0.032	15.1 ± 0.35
III	1.35 ± 0.037	7.4 ± 0.15	3.52 ± 0.081	8.2 ± 0.24	5.26 ± 0.156	13.1 ± 0.42
IV	1.44 ± 0.034	4.0 ± 0.09	3.75 ± 0.135	19.7 ± 0.55	5.53 ± 0.138	17.9 ± 0.54
V	0.02 ± 0.001	n.d.	0.05 ± 0.002	n.d.	0.05 ± 0.002	n.d.
VI	0.04 ± 0.002	n.d.	0.05 ± 0.002	n.d.	0.05 ± 0.002	n.d.
VII	0.01 ± 0.001	n.d.	0.02 ± 0.001	n.d.	0.03 ± 0.001	n.d.
VIII	1.68 ± 0.055	7.3 ± 0.23	2.39 ± 0.051	10.5 ± 0.21	2.85 ± 0.130	7.6 ± 0.21
IX	0.07 ± 0.002	n.d.	0.18 ± 0.005	n.d.	0.21 ± 0.011	n.d.

a Amount of α-(1→3)-glucan in mycelium dry mass. Results are shown as mean ± SD of three independent experiments.

n.d.: not detected.

**Table 4:** Effect of CWP prepared from selected mycelia of *L. sulphureus* CBS 388.61 on mutanase production by *T. harzianum* in shaken flask cultures^a^.

CWP		Mutanase activity	
	
Source	α-(1→3)-Glucan (%)	U/mL	U/g α-(1→3)-Glucan
1 week-old mycelium on medium:			
I	8.0 ± 0.23	0.063 ± 0.003	1.969 ± 0.058
II	4.3 ± 0.11	0.034 ± 0.001	1.976 ± 0.034
III	13.2 ± 0.32	0.030 ± 0.001	0.568 ± 0.020
IV	7.0 ± 0.21	0.063 ± 0.002	2.250 ± 0.087
VIII	12.0 ± 0.34	0.067 ± 0.003	1.396 ± 0.060
3 week-old mycelium on medium:			
I	16.2 ± 0.45	0.109 ± 0.004	1.682 ± 0.048
II	12.6 ± 0.24	0.074 ± 0.002	1.468 ± 0.066
III	15.6 ± 0.55	0.106 ± 0.003	1.699 ± 0.080
IV	27.8 ± 0.51	0.341 ± 0.011	3.067 ± 0.092
VIII	15.4 ± 0.33	0.119 ± 0.004	1.932 ± 0.056
5 week-old mycelium on medium:			
I	17.4 ± 0.53	0.115 ± 0.003	1.652 ± 0.052
II	22.0 ± 0.81	0.186 ± 0.006	2.114 ± 0.085
III	16.8 ± 0.47	0.129 ± 0.005	1.920 ± 0.086
IV	25.8 ± 0.88	0.293 ± 0.009	2.839 ± 0.125
VIII	12.0 ± 0.48	0.114 ± 0.003	2.375 ± 0.052

a Submerged cultures were performed on modified Mandels’ A medium with 0.4% CWPs in 500 mL conical flasks, each containing 100 mL of the medium. Initial medium pH was 5.3. Results are shown as mean ± SD of three independent experiments.
